# Evolution of *Parallel Spindles Like *genes in plants and highlight of unique domain architecture^#^

**DOI:** 10.1186/1471-2148-11-78

**Published:** 2011-03-24

**Authors:** Riccardo Aiese Cigliano, Walter Sanseverino, Gaetana Cremona, Federica M Consiglio, Clara Conicella

**Affiliations:** 1 CNR - National Research Council of Italy, Institute of Plant Genetics, Research Division Portici, Via Università 133, 80055 Portici. Italy; 2 DISSPAPA, Dept. of Soil, Plant and Environmental Sciences, University of Naples "Federico II", Via Università 100, 80055 Portici. Italy

## Abstract

**Background:**

Polyploidy has long been recognized as playing an important role in plant evolution. In flowering plants, the major route of polyploidization is suggested to be sexual through gametes with somatic chromosome number (2*n*). *Parallel Spindle1 *gene in *Arabidopsis thaliana *(*AtPS1*) was recently demonstrated to control spindle orientation in the 2nd division of meiosis and, when mutated, to induce 2*n *pollen. Interestingly, *AtPS1 *encodes a protein with a FHA domain and PINc domain putatively involved in RNA decay (i.e. Nonsense Mediated mRNA Decay). In potato, 2*n *pollen depending on parallel spindles was described long time ago but the responsible gene has never been isolated. The knowledge derived from *AtPS1 *as well as the availability of genome sequences makes it possible to isolate potato *PSLike *(*PSL*) and to highlight the evolution of *PSL *family in plants.

**Results:**

Our work leading to the first characterization of *PSLs *in potato showed a greater *PSL *complexity in this species respect to *Arabidopsis thaliana*. Indeed, a genomic *PSL *locus and seven cDNAs affected by alternative splicing have been cloned. In addition, the occurrence of at least two other *PSL *loci in potato was suggested by the sequence comparison of alternatively spliced transcripts.

Phylogenetic analysis on 20 *Viridaeplantae *showed the wide distribution of *PSLs *throughout the species and the occurrence of multiple copies only in potato and soybean.

The analysis of PSL^FHA ^and PSL^PINc ^domains evidenced that, in terms of secondary structure, a major degree of variability occurred in PINc domain respect to FHA. In terms of specific active sites, both domains showed diversification among plant species that could be related to a functional diversification among *PSL *genes. In addition, some specific active sites were strongly conserved among plants as supported by sequence alignment and by evidence of negative selection evaluated as difference between non-synonymous and synonymous mutations.

**Conclusions:**

In this study, we highlight the existence of PSLs throughout *Viridaeplantae*, from mosses to higher plants. We provide evidence that *PSLs *occur mostly as singleton in the analyzed genomes except in soybean and potato both characterized by a recent whole genome duplication event. In potato, we suggest the candidate *PSL *gene having a role in 2*n *pollen that should be deeply investigated.

We provide useful insight into evolutionary conservation of FHA and PINc domains throughout plant PSLs which suggest a fundamental role of these domains for PSL function.

## Background

Polyploidy represents the occurrence of more than two complete sets of chromosomes in an organism and has long been recognized as playing an especially important role in plant evolution [1]. In flowering plants, polyploidy extent has been largely underestimated in terms of its commonality. Indeed, major recent advances in genomic analysis has revealed that almost all angiosperms have experienced at least one round of whole genome duplication during their evolution.

The wide spreading of polyploidy throughout the angiosperms can be related to their highly plastic genome structure, as inferred from their tolerance to changes in chromosome number, genome size and epigenome [2]. Although information with regard to the modes of polyploidization is limited, the major route of polyploidization seems to be sexual through the functioning of gametes with somatic chromosome number (2*n *gametes) [3]. Indeed, sexual polyploidization as compared to asexual would explain better the success of polyploid species in terms of higher fitness and more genetic flexibility. The control of 2*n *gamete formation has been generally attributed to the action of single recessive genes. These genes exhibit incomplete penetrance and variable expression that is significantly influenced by genetic, environmental and developmental factors [4]. The molecular mechanisms leading to 2*n *gametes have only recently begun to be uncovered [5,6]. In particular, d'Erfurth and colleagues [7] isolated and characterized *Parallel Spindle1 *gene in *Arabidopsis thaliana *(*AtPS1*) that controls diploid pollen formation through spindle orientation in the second division of meiosis. The occurrence of parallel spindles at meiosis II is a frequently found mechanism for 2*n *pollen formation that was described in potato many decades ago [8,9]. In potato, *ps *mutants have been used for breeding purposes in order to introgress beneficial traits from diploid (2*n *= 2*x *= 24) relatives into cultivated strains [10]. However, the gene *ps *leading to 2*n *pollen via parallel spindles was not isolated, so far.

Interestingly, AtPS1 is a protein which contains contemporarily a ForkHead Associated domain (FHA), and a C-terminal PilT N-terminus domain (PINc). So far, the FHA domain has been found in more than 5600 different proteins from prokaryotes to higher eukaryotes involved in several processes including cell cycle control, DNA repair, protein degradation, transcription and pre-mRNA splicing [11]. FHA domain was shown to recognize phosphothreonine-containing epitopes [12]. PINc domain has been found in more than 3600 proteins in all life kingdoms. PINc domain has RNA nuclease activity [13]. In eukaryotes, PINc-containing proteins, such as human SMG6 and SMG5, were linked to Nonsense-Mediated mRNA Decay (NMD), that recognizes and rapidly degrades mRNAs containing Premature translation Termination Codons (PTCs).

In this study, a sequence-homology-based strategy was carried out to isolate *PS *gene from a diploid potato. Through this approach, a genomic locus *PS-Like *(*PSL*) and seven cDNAs affected by alternative splicing have been cloned. The occurrence of at least two other *PSL *loci is suggested in potato. In order to shed light on the evolution and function of *PSL *genes in plants, a phylogenetic analysis of *PSL *genes was performed and FHA/PINc domains were compared. As far as we know, this is the first report about the isolation and characterization of *PSL *in a crop. We also demonstrate the conservation of this gene family throughout plants.

## Results

### Cloning and characterization of *PSL *genes in potato

**Figure 1:**
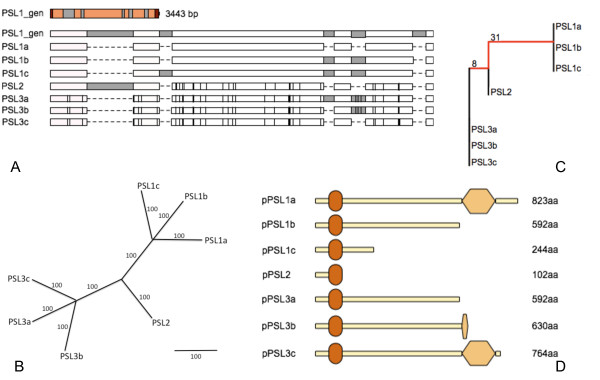
**Potato *PSL *gene family**. (A) PSL1 gene structure (*PSL1_gen*) with UTRs reported in red, exons in orange and introns in grey is showed together with the schematic alignment of the cloned cDNAs and *PSL1_gen *sequences, with exons in white, introns in grey and nucleotide differences respect to *PSL1_gen *as vertical black lines. (B) Maximum Likelihood phylogenetic tree of *PSL *cDNAs is reported with bootstrap values for each node and branch lengths measured in bootstrap values. (C) The distance in terms of number of different nucleotides is reported for the cloned *PSL *cDNAs. (D) Schematic representation of PSL predicted proteins obtained after *in silico *translation of the corresponding cDNAs showing FHA and PINc domains as ovals and exagons, respectively.

Using the predicted protein sequence of *AtPS1*, two ESTs were identified on DFCI Potato Gene Index (http://compbio.dfci.harvard.edu/tgi) named TC194262 and DN590600 corresponding to FHA and PINc domains, respectively. The primers designed on the above mentioned ESTs allowed to isolate in diploid potato a 3 Kbp genomic clone (*PSL1*) lacking UTR regions. In order to complete genomic sequence of *PSL1*, by querying Potato Genome Sequencing Consortium Database (http://www.potatogenome.net/index.php) and SOL Genomic Network (http://solgenomics.net) we retrieved a 3 Kbp region of *S. phureja *v3 scaffold PGSC0003DMS000001829 (*phuPSL*) and a tomato BAC clone AC211085.1 (*SlPSL1*) sharing 97% and 93% of sequence identity with *PSL1*, respectively. Primers designed on the tomato sequence were used to isolate a potato 5.3 Kbp *PSL1 *genomic region spanning 2 Kbp upstream of the start codon to about 200 bp downstream of the stop codon [GenBank:HQ418834]. *In silico *gene prediction showed a structure of *PSL1 *composed by six exons and five introns (Figure 1A) and a 2.4 Kbp hypothetical *PSL1 *cDNA (*PSL1*_pred) with a GC content of 42% encoding a 92.5 kDa protein of 823 aminoacids (pPSL1_pred).

Given the meiotic function of *AtPS1*, potato *PSL1 *cDNA was isolated from pre-bolting buds. Seven different cDNAs ranging from 2.3 to 2.7 Kbp were isolated including *PSL1a *of 2.4 Kbp corresponding to *PSL1*_pred. The sequence alignment between cDNAs and the genomic *PSL1 *indicated the presence of different groups of related *PSL *sequences encoded by more than one locus (Figure 1A). In order to investigate the relationship between the different *PSL *cDNAs, we conducted a phylogenetic analysis that suggested the existence of three different loci named *PSL1*, *PSL2 *and *PSL3 *(Figure 1B). On the basis of sequence similarity, we assigned *PSL1a*, *PSL1b *and *PSL1c *cDNAs [GenBank:HQ418835, GenBank:HQ418836 and GenBank:HQ418837] to genomic *PSL1*, *PSL3a*, *PSL3b *and *PSL3c *cDNAs [GenBank:HQ418839, GenBank:HQ418840 and GenBank:HQ418841] to *PSL3 *and the last cDNA to *PSL2 *[GenBank:HQ418838]. Moreover, the distance measured as the number of different nucleotides among the seven cDNAs showed that *PSL2 *was more similar to *PSL3 *than to *PSL1 *(Figure 1C). The nucleotide comparison between the predicted *phuPSL *cDNA and *PSL1a*, *PSL2 *and *PSL3c *cDNAs showing a similarity of 98.5%, 99.1% and 99.6%, respectively, suggested that *phuPSL *locus corresponds to *PSL3 *being the differences explained by the different genetic background.

Based on the sequences of cloned *PSL *cDNAs, *PSL1b *and *PSL1c*, *PSL3a *and *PSL3b *are alternative splicing forms of *PSL1 *and *PSL3 *since they retained complete or partial introns causing the formation of premature stop codons (PTCs) (Figure 1A). Moreover, the cloned *PSL2 *cDNA showed a PTC caused by the retention of the second intron. As a consequence, all the predicted PSL proteins, except pPSL1a and pPSL3c, had truncated or lacking PINc domain (Figure 1D).

In order to evaluate whether the alternative splicing *PSL *variants were possible target of degradation through NMD we calculated the distance between PTCs and the successive exon-exon junction. Being this distance more than 50-55 nt according to Nagi and Maquat [14] we could consider *PSL1b, PSL1c, PSL2*, *PSL3a *and *PSL3b *as target of NMD.

### Phylogenetic analysis of *PSL *genes in sequenced *Viridaeplantae*

In order to investigate the evolution of *PSL *family, a phylogenetic analysis was performed by a search on Interpro (http://www.ebi.ac.uk/interpro) for proteins with both FHA and PINc domains. Interestingly, these domains were contemporary present only in plants, except for the multidrug-efflux transporter NAEGRDRAFT_78193 from the amoeboid *Naegleria gruberi*.

**Figure 2:**
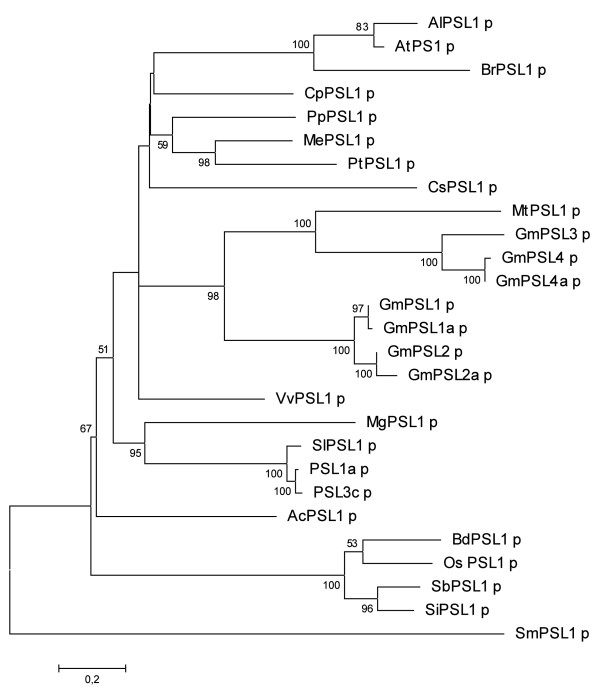
**Phylogenetic tree of plant PSL proteins**. Maximum Likelihood phylogenetic tree of PSL predicted proteins from 20 plant species. Bootstrap values are shown for each node. The tree is drawn to scale, with branch lengths measured in the number of substitutions per site. At = *Arabidopsis thaliana*; Ac = *Aquilegia coerulea*; Al = *Arabidopsis lyrata*; Bd = *Brachypodium distachyon*; Br = *Brassica rapa*; Cp = *Carica papaya*; Cs = *Cucumis sativus*; Gm = *Glycine max*; Me = *Manihot esculenta*; Mg = *Mimulus guttatus*; Mt = *Medicago truncatula*; Os = *Oryza saliva*; Pp = *Prunus persica*; Pt = *Populus trichocarpa*; Sb = *Sorghum bicolour*; Si = *Setaria italica*; Sl = *Solanum lycopersicon*; Sm = *Selaginella moellendorffii*; Vv = *Vitis vinifera*.

**Figure 3:**
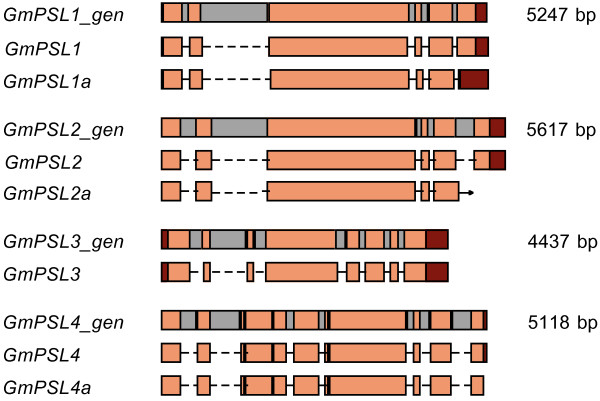
**Alternative splicing of *GmPSL *genes**. *GmPSL1_gen*, *GmPSL2_gen*, *GmPSL3_gen*, *GmPSL4_gen *gene structures are reported with UTRs in red, exons in orange and introns in grey. The mRNAs are reported with UTRs in red, exons in orange and spliced introns as interrupted lines underneath each gene structure. Arrow indicates the lack of complete sequence.

PSL1a sequence was blasted against the Phytozome v6 database (http://www.phytozome.net) that contains the genomic sequences of 22 organisms and against the SOL Genomics Network containing the tomato genome assembly. Afterwards, 25 sequences of predicted PSL proteins were collected from 19 different organisms (Additional File 1), since *Physcomitrella patens*, *Ricinus communis*, *Volvox carteri*, *Zea mays *and *Chlamydomonas rehinardtii *seemingly lack *PSL *genes. The sequences were then aligned and the Maximum-Likelihood phylogenetic tree is shown in Figure 2. The distribution of PSLs is in agreement with the known phylogenetic relationships between species among dicots and monocots. Moreover, one *PSL *locus was found in the analyzed plant species, except for *Glycine max*. Indeed, four different *PSL *loci were identified in soybean and three of them encode alternative transcripts. Differently from potato, the alternative transcripts of soybean retain PINc domain being the splicing sites located at the 3'-end of mRNA (Figure 3).

### Analysis of FHA and PINc secondary structure and active sites in PSL proteins

**Figure 4:**
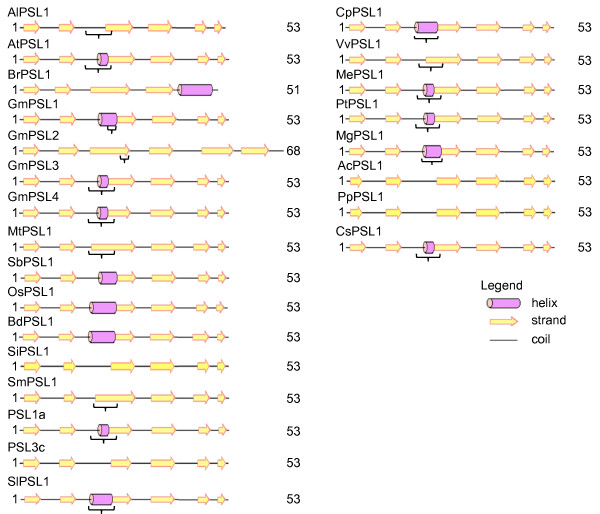
**Predicted secondary structures of PSLs^FHA^**. FHA domain predicted secondary structures of PSLs are reported with alpha-helices as violet tubes, beta-strands as yellow arrows and coiled regions as lines. Regions underlined with brackets show low significance as estimated by PSIPRED. The domain length is also reported. At = *Arabidopsis thaliana*; Ac = *Aquilegia coerulea*; Al = *Arabidopsis lyrata*; Bd = *Brachypodium distachyon*; Br = *Brassica rapa*; Cp = *Carica papaya*; Cs = *Cucumis sativus*; Gm = *Glycine max*; Me = *Manihot esculenta*; Mg = *Mimulus guttatus*; Mt = *Medicago truncatula*; Os = *Oryza saliva*; Pp = *Prunus persica*; Pt = *Populus trichocarpa*; Sb = *Sorghum bicolour*; Si = *Setaria italica*; Sl = *Solanum lycopersicon*; Sm = *Selaginella moellendorffii*; Vv = *Vitis vinifera*.

**Figure 5:**
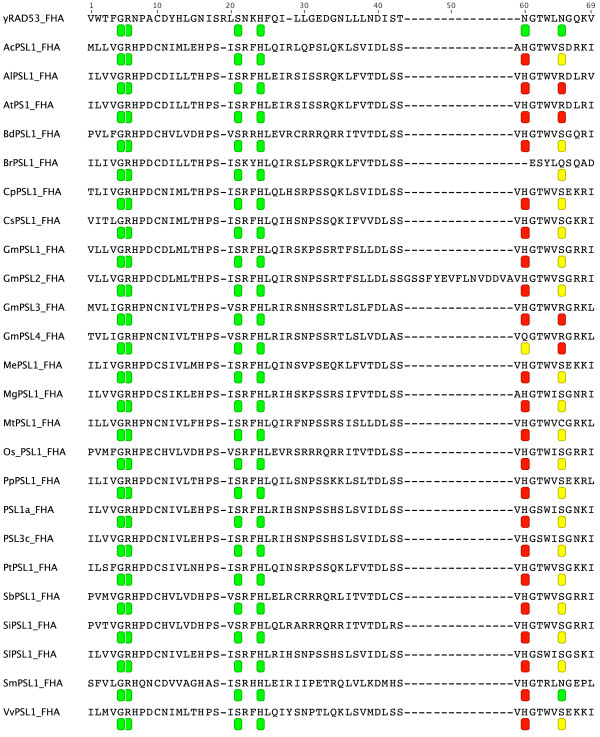
**Alignment of PSLs^FHA ^and yRAD53p^FHA^**. The protein domain alignment of PSLs^FHA ^and yRAD53p^FHA ^is reported showing the conservation of active sites labeled with a green octagon when the residues are conserved. A yellow or a red octagon mark an aminoacid substitution of same or different polarity, respectively. At = *Arabidopsis thaliana*; Ac = *Aquilegia coerulea*; Al = *Arabidopsis lyrata*; Bd = *Brachypodium distachyon*; Br = *Brassica rapa*; Cp = *Carica papaya*; Cs = *Cucumis sativus*; Gm = *Glycine max*; Me = *Manihot esculenta*; Mg = *Mimulus guttatus*; Mt = *Medicago truncatula*; Os = *Oryza saliva*; Pp = *Prunus persica*; Pt = *Populus trichocarpa*; Sb = *Sorghum bicolour*; Si = *Setaria italica*; Sl = *Solanum lycopersicon*; Sm = *Selaginella moellendorffii*; Vv = *Vitis vinifera*.

In order to assess the conservation degree of PSLs, we predicted and compared the secondary structure of FHA and PINc domains using the SMART (http://smart.embl-heidelberg.de) and PSIPRED (http://bioinf.cs.ucl.ac.uk/psipred) tools. It is reported that FHA domain is 80-100 aminoacid (aa) long folded into a 11-stranded beta sandwich, which sometimes contains small helical insertions between the loops connecting the strands. However, *in silico *analysis of FHA displays only 8 beta-strands (b-strands) out of 11 including the residues involved in phosphopeptide recognition and stabilisation of domain architecture [15]. Using the above mentioned tools on yeast RAD53p [NCBI:6325104], a well characterized FHA containing protein [12], the identified FHA region was 52 aa covering 6 beta-strands (data not shown). As shown in Figure 4, the length of the predicted FHA region in our dataset was 53 aa except for *Brassica rapa *(51 aa) and for *Glycine max *PSL2 (68 aa). While the majority of FHA domains showed 6 beta-strands, *Brassica rapa *FHA was predicted to be composed of 4 consecutive beta-strands followed by an alpha helical region. The group of monocots, *Brachypodium distachyon*, *Oryza sativa *and *Sorghum bicolor*, as well as dicot *Glycine max *(*GmPSL1*) showed a helical insertion between the 2nd and the 3rd beta-strand. In other species this helical insertion was predicted but at a low confidence value as estimated by PSIPRED. Afterward, we compared the active sites in yRAD53p with those present in PSL^FHA ^domains through the protein alignment showed in Figure 5. It can be observed that glycine-5, arginine-6, serine-21 and histidine-24 in FHA domain are perfectly conserved. The arginine-19 seems to be absent in all plant sequences. In the analyzed species, asparagine-60 showed a substitution with histidine, characterized by a different polarity, except for soybean PSL4 glutamine-60 and *Brassica rapa *that seems to lack this site. Asparagine-66 is mostly substituted with the similar polar serine. Instead, AlPSL1, AtPSL1, GmPSL3 and GmPSL4 exhibited arginine-66 with different polarity while BrPSL1 glutammine-66 and MtPSL1 cysteine-66 both showing similar polarity of asparagine.

**Figure 6:**
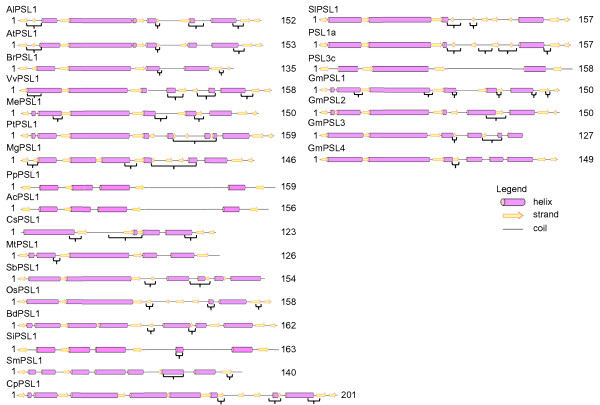
**Predicted secondary structure of PSLs^PINc^**. PINc domain predicted secondary structures of PSLs are reported with alpha-helices as violet tubes, beta-strands as yellow arrows and coiled regions as lines. Regions underlined with brackets show low significance as estimated by PSIPRED. The domain length is also reported. At = *Arabidopsis thaliana*; Ac = *Aquilegia coerulea*; Al = *Arabidopsis lyrata*; Bd = *Brachypodium distachyon*; Br = *Brassica rapa*; Cp = *Carica papaya*; Cs = *Cucumis sativus*; Gm = *Glycine max*; Me = *Manihot esculenta*; Mg = *Mimulus guttatus*; Mt = *Medicago truncatula*; Os = *Oryza saliva*; Pp = *Prunus persica*; Pt = *Populus trichocarpa*; Sb = *Sorghum bicolour*; Si = *Setaria italica*; Sl = *Solanum lycopersicon*; Sm = *Selaginella moellendorffii*; Vv = *Vitis vinifera*.

**Figure 7:**
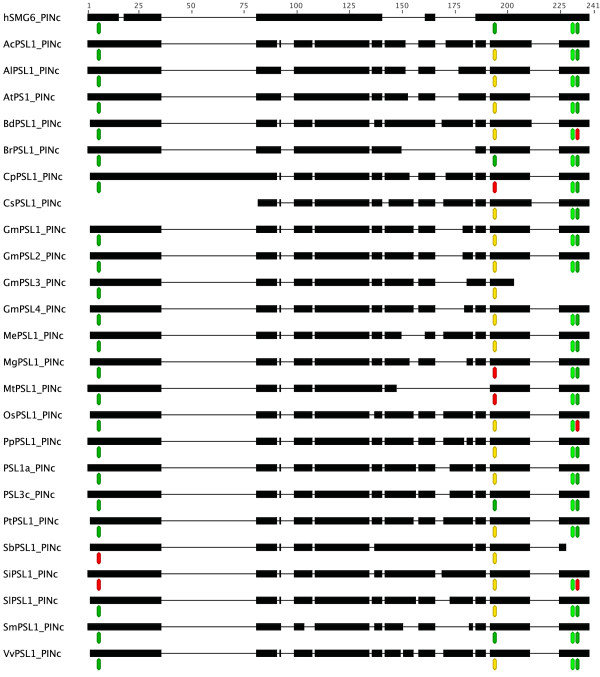
**Comparison of catalytic residues among PSLs^PINc ^and hSMG6^PINc^**. A schematic protein domain alignment of PSLs^PINc ^and hSMG6^PINc ^is reported showing the conservation of catalytic residues. Black lines represent gaps in the alignment. Active sites are labeled with a green octagon when the residues are conserved among PSLs^PINc ^and hSMG6^PINc^. A yellow or a red octagon mark an aminoacid substitution of same or different polarity, respectively. At = *Arabidopsis thaliana*; Ac = *Aquilegia coerulea*; Al = *Arabidopsis lyrata*; Bd = *Brachypodium distachyon*; Br = *Brassica rapa*; Cp = *Carica papaya*; Cs = *Cucumis sativus*; Gm = *Glycine max*; Me = *Manihot esculenta*; Mg = *Mimulus guttatus*; Mt = *Medicago truncatula*; Os = *Oryza saliva*; Pp = *Prunus persica*; Pt = *Populus trichocarpa*; Sb = *Sorghum bicolour*; Si = *Setaria italica*; Sl = *Solanum lycopersicon*; Sm = *Selaginella moellendorffii*; Vv = *Vitis vinifera*.

The analysis of PINc domain in our dataset started with the prediction of its secondary structure in human SMG6 (hSMG6) [UniProt:Q86US8]. It is reported that hSMG6^PINc ^(hSMG6) is 182 aa folded into a 5-stranded parallel beta-sheets that is highly twisted and alpha-helices arranged on both sides of each beta-sheet for a total of 6. Three aspartate residues are essential for PINc activity in hSMG6, while a threonine or a serine in the sequence (T/S)XD might be involved in catalytic role on the basis of similarity with other PINc domains [16,17]. SMART predicted a PINc domain of 152 aa lacking the first and the last alpha-helices while PSIPRED predicted 4 beta-sheets and 4 alpha helices in hSMG6 (data not shown). In our dataset the PINc domain ranged from a minimum of 123 aa in *Cucumis sativus *to a maximum of 162 aa in *Brachypodium distachyon *(Figure 6). In our prediction, the number of beta-sheets ranged from 3 in GmPSL3 and CsPSL1 to 9 in CpPSL1. The number of predicted alpha-helices ranged from 3 in CsPSL1, OsPSL1 and PSL3c to 7 in BdPSL1 and CpPSL1. Afterward, we compared the active residues of PINc in PSL proteins. The alignment between PINc domain of PSL proteins and hSMG6 is shown in Figure 7 (refer to Additional file 2: Alignment of PSL^PINc ^and hSMG6^PINc ^residues to look at raw alignment). BrPSL1, SmPSL1 and PSL3c show the three expected aspartate residues at positions 6, 194 and 233 of the alignment. SiPSL1 and SbPSL1 have a substitution of two aspartate residues with asparagine-6, glutammate-194. When a substitution occurs, the aspartate-194 is mostly replaced with glutammate-194 except for CpPSL1 showing a different polarity residue (lysine-194) and MgPSL1 showing an alyphatic residue (alanine-194). The aspartate-233 is widely conserved except in BdPSL1, OsPSL1 and SiPSL1 where it is substituted with a serine-233. Most of the PSL proteins show a catalytic serine-231 in the described pattern SXD (where X can be D, N, E or S in this study) instead of threonine-231 in hSMG6, PtPSL1, SiPSL1, SmPSL1 and VvPSL1. GmPSL3 and SbPSL1 are the only proteins lacking the C-terminal extremity of PINc interrupting at leucine-203 and lysine-227 respectively thus missing the catalytic threonine/serine-231 and the aspartate-233.

### Selective pressure among amino acid sites in the *PSL *family

**Figure 8:**
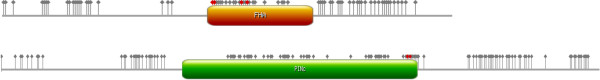
**Distribution of negatively selected codons in PSL^FHA ^and PSL^PINc^**. A schematic representation of FHA and PINc domains is reported showing negatively selected sites as grey diamonds. Red diamonds mark active sites subjected to negative selection.

In order to test for presence of positive selection at individual amino acid codons, the site specific models implemented in DataMonkey 2010 webserver (http://www.datamonkey.org) [18] were used. The Integrative Selection Analysis of FEL, SLAC and FER alghoritms evidenced no significant positive selected sites (dN-dS > 0). Conversely, 217 significant negative selected sites (dN-dS < 0) were identified. The region between FHA to PINc domains included few negative selected sites which were mostly located near the functional domains. The codons encoding the highly conserved active sites glycine-5, arginine-6, serine-21 and histidine-24 in FHA and aspartate-6, serine/threonine-231 and aspartate-233 in PINc were also subjected to negative selection (Figure 8). These observations support the results obtained through sequence alignment and evidence an occurrence of negative pressure upon non-synonymous mutations in *PSL *genes. In particular, the regions next to the functional domains and the codons for active sites were affected.

## Discussion

In this work, we isolated PSL genes in a diploid potato (2*n *= 2*x *= 24) and through *in silico *analysis we identified PSL in other plant species. The function of PSL in plants can be inferred from *Arabidopsis *study on *AtPS1 *gene that appears responsible for the spindle orientation at meiosis II playing a regulatory function, likely via RNA decay [7].

In all the analysed species, except soybean and potato, *PSL *loci appear as singleton behaving as resistant to duplication. It is known that angiosperms are mostly paleopolyploids [19], many having survived multiple duplication events [20]. Analysis of genome sequences shows that some genes duplicate and persist as multiple copies after whole genome duplication (WGD) while other genes are iteratively returned to singleton status. Moreover, it seems that the singleton status is consistently restored for some functional gene groups like those involved in DNA repair or signal transduction [21]. The evolutive history of potato and soybean could explain the expansion of *PSL *genes occurring in these species that have experienced recent WGD. In cultivated potato (2*n *= 4*x *= 48), WGD is reported to have occurred about 10 million of years ago (*mya*) after the divergence from *Solanum lycopersicum *[22]. In soybean (2*n *= 2*x *= 40), which seems to be an ancient allopolyploid on the basis of two different centromeric repeat classes [23], two rounds of WGD happened the latter aging 10-15 *mya *[24,25]. In addition, the location of the four soybean *PSLs *on different chromosomes reinforce their origin from WGD rather than tandem duplications.

The evident feature of PSL proteins is the contemporary presence of FHA and PINc domains. FHA is reported as a phosphothreonine (pT) binding domain showing a 11 beta-sandwich secondary structure, also containing small helical insertions between the beta-strands [15]. The FHA active sites are usually located in the loops connecting b3/b4, b4/b5 and b6/b7 strands. RAD53p^FHA ^arginine-70, serine-85 and asparagine-107 (corresponding to arginine-6, serine-21 and asparagine-60 in Figure 5) are involved in the interaction with the phosphopeptide backbone. Arginine-83 (corresponding to arginine-19 in Figure 5) is the most important aminoacid for FHA binding specificity. Indeed, its conversion to glycine shifts the binding from pTXXD to pTXXI peptides in RAD53p^FHA ^interaction experiments using surface plasmon resonance [15]. Glycine-69 and histidine-88 (corresponding to glycine-5 and histidine-24 in Figure 5) stabilize the architecture of the binding site. The remaining conserved residue, asparagine-112 (corresponding to asparagine-65 in Figure 5), is remote from the peptide binding site and serves to tether the beta turn between b7/8 to b10 [12,15]. The comparison of predicted secondary structure in our dataset showed a wide conservation among plant species regarding the number and the position of the beta-sheets and the presence of an helical insertion between the second and the third beta-sheet as a characteristic feature of monocot PSL proteins. The comparison of active sites showed that two PSL^FHA ^residues involved in pT binding as well those involved in stabilisation of the architecture of the binding site are fully conserved except for asparagine-65 that is replaced with a different polarity residue (arginine) in AlPSL1, AtPS1, GmPSL3 and GmPSL4 with unknown possible effect on domain architecture. Moreover, as compared to RAD53p^FHA^, a conserved substitution in plants is a histidine instead of asparagine-60, known to be important for the selectivity of binding of phosphothreonine upon phosphoserine. The different polarity between these two residues might suggest a functional diversification of this active site. However, the consequences of this substitution in PSL^FHA ^domain in terms of ligand binding cannot be easily predicted and should be assessed by protein:protein interaction analysis. It is also intriguing the lack of arginine-19 in plants raising the question of binding selectivity for plant PSL^FHA^.

As regards PINc domain, we made reference to human SMG6^PINc ^which has RNAse activity and it is composed of alternating beta-sheets and alpha-helices. It is reported that hSMG6 is involved in NMD together with hSMG5 and hSMG7 [25]. In Arabidopsis, *AtSMG7 *was proved to be involved in NMD and to be required for meiotic spindle organization in meiosis II [26]. As reported by Glavan and colleagues [17], hSMG6 and other PINc domains show three conserved aspartic residues at positions 1251, 1353 and 1392 (corresponding to aspartate-6, -194 and -233 in Additional File 2) involved in Mg^2+ ^binding. Threonine or serine embedded in the motif (T/S) XD is proposed to be the catalytic site on the basis of sequence alignment of PIN domains and it is located at residue-1390 (corresponding to threonine-231 in Figure 7) in hSMG6^PINc^.

Our results showed differences of secondary structures in PSL^PINc ^domains even between phylogenetically close organisms. Moreover, the typical alternation between beta-sheets and alpha-helices does not seem to be respected. In spite of these differences, the active sites showed a wide conservation among plants. The aspartate-6 is conserved in all proteins except for SbPSL1 and SiPSL1 where an asparagine is present. This substitution maybe occurred after the divergence of BEP from PACCAD clades among *Poaceae *given its absence in *Oryza sativa*, *Brachypodium distachyon *and *Selaginella moellendorffii*. The residue-194 showed the major degree of variation among the species mostly characterized by glutamate instead of aspartate. This substitution is not related to phylogenesis, being glutamate present in both monocots and dicots while aspartate-194 is shared by the *Brassicaceae *BrPSL1, the *Selaginellaceae *SmPSL1 and the *Solanaceae *PSL3c. In *Carica papaya*, *Mimulus guttatus *and *Medicago truncatula *the change involved aminoacids with a different polarity such as lysine, alanine, and asparagine. The aspartate-233 is widely conserved among PSL proteins, except those of *Poaceae *OsPSL1, SiPSL1 and BdPSL1 showing a serine-233, likely a substitution occurred after the separation of monocots from dicots. GmPSL3 and SbPSL1 lack this residue due to PINc domain truncation at leucine-203 and lysine-227, respectively. As compared to hSMG6, the majority of plants showed a serine instead of threonine-231 that is present only in PtPSL1, SiPSL1, SmPSL1 and VvPSL1. This substitution should not compromise the PINc activity since serine and threonine are the most represented residues at this position in PINc domains of different organisms [16]. Based on the evidence that the three aspartate residues of PINc domain are crucial for RNase activity, we can argue that PSLs lacking one of these residues or showing aminoacid with different polarity (BdPSL1, CpPSL1, CsPSL1, GmPSL3, MgPSL1, MtPSL1, SbPSL1, SiPSL1, OsPSL1) have no enzymatic activity. However, we cannot exclude that they are partners of other proteins retaining RNAse activity. Indeed, in human, it is known that hSMG5 lacking two aspartate residues respect to hSMG6 has no enzymatic activity but the interaction between hSMG5 and hSMG7 led to a functional nuclease activity of SMG7-SMG5 complex [17].

Functional analysis of *Arabidopsis PS1 *reinforced the evidence that the defects in meiotic spindle orientation in meiosis II led to the formation of diplopollen. Among the analysed species, *Manihot esculenta *(2*n *= 2*x *= 36) was reported to produce 2*n *pollen but the cytological mechanisms underlying its formation were not deeply investigated [27]. Since the predicted MePSL protein has FHA and PINc active sites similar to those of other species, it is likely that the mechanism leading to 2*n *pollen in *Manihot esculenta *does not involve parallel spindles.

In the potato genotype analysed in this study, neither spindle defects nor 2*n *pollen have been reported [28]. In this genotype, we identified three *PSL *loci and seven transcripts. Based on AtPS1 characterized by FHA and PINc domains we can suspect that PSL1a and PSL3c, carrying both domains, are functional proteins. In addition, it can be speculated that PSL1a that evidences the same PINc active residues of AtPS1 is the strongest candidate for the regulation of spindle orientation in meiosis II. The landscape of alternative splicing in potato PSL is not surprising since it has been already observed for genes involved in *Arabidopsis *meiosis. For instance, *AtSPO11-1*, involved in double strand breaks (DSBs) required for meiotic recombination, exhibits up to ten splicing forms showing PTCs [29]. As inferred for *AtSPO11-1, PSLs *are possible target of NMD that could act as a post-transcriptional regulatory pathway for the proper expression of PSL. Alternative splicing was observed also in *Glycine max *but the lack of PTCs exclude NMD regulation of PSL transcripts. Defining the ligands of FHA and PINc domains and proving PSL as a component in NMD are essential to link PSLs to plant evolution by polyploidization via 2*n *gametes.

## Conclusions

In this study, we show that PSLs are common genes across *Viridaeplantae*. We provide evidence that *PSLs *occur mostly as singleton in the analyzed genomes except in soybean and potato both characterized by a recent event of whole genome duplication. We provide useful insight into evolutionary preservation of FHA and PINc domains throughout plant PSL genes, suggesting a fundamental role of these domains for PSL function. FHA appeared to be highly conserved, while PINc secondary structure and specific active sites showed a less conserved landscape, suggesting a functional diversification among PSL genes.

## Methods

### Plant material

A previously described [30] diploid clone of potato (named T710) coming from hybridization between *Solanum tuberosum *haploid USW3304 (2*n *= 2*x *= 24) and *S. chacoense *(2*n *= 2*x *= 24) has been used to isolate *PSL *genes.

### Potato *PSL *cloning and sequence analysis

Plant genomic DNA was isolated from leaves using the DNeasy Plant Mini Kit (QIAGEN http://www1.qiagen.com). Bacterial plasmid DNA was isolated using QIAprep Spin Miniprep Kit (QIAGEN). Total RNA from prebolting buds was extracted using the RNeasy Plant Mini Kit (QIAGEN) and then treated with DNase I (Invitrogen, http://www.invitrogen.com) to remove residual genomic DNA. Primer pairs for cloning designed with PRIMER3 (http://frodo.wi.mit.edu/cgi-bin/primer3/primer3.cgi) are listed in Additional file 3: Primers used in this study.

*PSL1 *genomic fragment was amplified by PCR with primers PS_F1 and PS_R1 and full sequence with primers PS_F2 and PS_R2. The coding region of *PSL *transcripts was amplified by RT-PCR by using SuperScriptTM III Reverse Transcriptase (Invitrogen) with an oligo- dT_12-18 _(Invitrogen). The cDNA was subjected to PCR with PS_CF and PS_CR primers (Additional File 3). PCR products have been cloned into PCR2.1 with T/A Cloning kit (Invitrogen). Nucleotide sequencing was carried out by Eurofins MWG Operon sequencing service (Germany).

*PSL1 *gene structure and cDNA predictions were carried out using FGENESH online tool (http://linux1.softberry.com/berry.phtml?topic=fgenesh&group=programs&subgroup=gfind) selecting Tomato as organism. *In silico *translation of cDNA sequences was carried out using the Expasy Translation tool (http://www.expasy.ch/tools/dna.html).

### Identification of unannotated *PSL *genes

The PSL protein sequences were identified and collected by TBLASTN [31] search against the Phytozome v6 database (http://www.phytozome.net/), SGN (SOL Genomic Network, http://sgn.cornell.edu/) and Potato Genome Sequencing Consortium (PGSC, http://potatogenomics.plantbiology.msu.edu/index.php?p=blast).

### Molecular Phylogenetic analysis by Maximum Likelihood method

The PSL protein sequences were firstly aligned by MUSCLE [32] using the default settings of MEGA5 [33]. The best model for Maximum Likelihood phylogeny analysis was chosen testing all the available models in ProtTest version 2.4 [34] with slow optimization strategy and selecting the one with highest AICc value. The evolutionary history was then inferred by using the Maximum Likelihood method according to Jones et al. w/freq. model [35]. The bootstrap consensus tree was inferred from 100 replicates [36]. Initial tree(s) for the heuristic search were obtained automatically as following. When the number of common sites is <100 or less than one fourth of the total number of sites, the maximum parsimony method was used, otherwise BIONJ method with MCL distance matrix was used. A discrete Gamma distribution was used to model evolutionary rate differences among sites (4 categories, +G, parameter = 1.3900). The rate variation model allowed for some sites to be evolutionarily invariable ([+I], 9.0566% sites). All ambiguous positions were removed for each sequence pair. Evolutionary analyses were conducted in MEGA5.

### PSL domain analysis

The locations of FHA and PINc domains within *PSL *genes were detected using SMART [37]. Secondary structure prediction was performed using domains from PSL sequences as input into the PSIPRED secondary structure prediction server [38]. The program MUSCLE [32] was used to do multiple sequence alignments of FHA and PINc domains in PSL proteins, yRAD53p [NCBI:6325104] and hSMG6 [UniProt:Q86US8]. Conservation of phosphothreonine-binding residues in FHA and of RNAse activity residues in PINc were determined by alignment with yRAD53p and hSMG6, respectively.

### Comparison of dN-dS values between *PSL *sequences

The coding sequences of PSLs were obtained from the databases reported in Additional File 1. The terminal codon was manually removed, then the codon alignment was performed by MUSCLE [32] using the default settings of MEGA5 [33] (Tamura *et al.*, personal communication). To select the best substitution model we used JmodelTest 0.1.1 [39,40] with default settings. The GTR [41] model was selected as the best fitting as evidenced by AICc values.

The codon alignment was then uploaded on DataMonkey 2010 server [18,42] and dN-dS evaluated using GTR as substitution model, and SLAC (default settings except for Global dN/dS value = estimated with CI), FEL and REL [43] alghoritms. Codons subjected to evolutive pressure were identified with Integrative Selection Analysis selecting significance levels for SLAC p < 0.1, FEL p < 0.1 and REL Bayes Factor < 50. Only codons with a significant dN-dS value according to all the three methods were reported.

## Authors' contributions

RAC performed the molecular research and the protein domain analysis, participated in phylogenetic analysis, study design and drafted the manuscript. WS carried out the phylogenetic analysis and participated in bioinformatic analysis design. GC participated in molecular analysis. FMC performed the molecular analysis design and revised the manuscript. CC conceived of and coordinated the study, and revised the manuscript. All authors read and approved the final manuscript.

## Note

#Contribution n 358 from CNR - National Research Council of Italy, Institute of Plant Genetics, Research Division Portici.

## Supplementary Material

Additional file 1 **PSL sequences used in this study**. Description of sequence names, accession numbers, organisms and source databases used in this study. Click here for file

Additional file 2 **Alignment of PSL^PINc ^and hSMG6^PINc ^residues**. The protein domain alignment of PSLs^PINc ^and hSMG6^PINc ^is reported showing the conservation of catalytic residues. Dotted lines represent gaps in the alignment. Active sites are labeled with a green octagon when the residues are conserved among PSLs^PINc ^and hSMG6^PINc^. A yellow or a red octagon mark an aminoacid substitution of same or different polarity, respectively. Click here for file

Additional file 3 **Primers used in this study**. Primers used for the isolation of *PSL *genomic clone and cDNAs Click here for file
