# Supplementary material for: Evolution of Parallel Spindles Like genes in plants and highlight of unique domain architecture#
Source: BMC Evol Biol. 2011 Mar 24;11:78. doi: 10.1186/1471-2148-11-78 (PMC3071787; doi:10.1186/1471-2148-11-78)
Supplement: Additional file 1 — PSL sequences used in this study. Description of sequence names, accession numbers, organisms and source databases used in this study. [file 1471-2148-11-78-S1.PDF]

Table 1 - PSL sequences used in this study

Species	Common Name	Database	Accession	Protein abbreviation
DICOTS				
Fabids				
Cucumis sativus	Cucumber	Phytozome	Cucsa.079920.1	CsPSL1
Glycine max	Soybean	Phytozome	Glyma02g08210.1	GmPSL1
Glycine max	Soybean	Phytozome	Glyma02g08210.2	GmPSL1a
Glycine max	Soybean	Phytozome	Glyma10g35930.1	GmPSL4
Glycine max	Soybean	Phytozome	Glyma10g35930.2	GmPSL4a
Glycine max	Soybean	Phytozome	Glyma16g27290.1	GmPSL2
Glycine max	Soybean	Phytozome	Glyma16g27290.2	GmPSL2a
Glycine max	Soybean	Phytozome	Glyma20g31660.1	GmPSL3
Manihot esculenta	Manioc	Phytozome	cassava24482.valid.m1	MePSL1
Medicago truncatula	Barrel medic	Phytozome	Medtr1g122320.1	MtPSL1
Prunus persica	Peach	Phytozome	ppa000455m.g	PpSPL1
Populus trichocarpa	Black cotton wood	GeneBank	XM_002327195.1	PtPSL1
Lamiids				
Mimulus guttatus	Monkey-flower	Phytozome	mgf021378m	MgPSL1
Solanum lycopersicum	Tomato	SGN	SL1.00sc02597_37.1.1	SlPSL1
Malvids				
Arabidopsis thaliana	Thale cress	AGI	At1g34355.1	AtPS1
Arabidopsis lyrata	-	GeneBank	XM_002891053.1	AlPSL1
Brassica rapa	Field mustard	Phytozome	AC232513_fgenesh	BrPSL1
Carica papaya	Papaw	Phytozome	evm.model.supercontig_66.57	CpPSL1
Rosids incertae sedis				
Vitis vinifera	Grape	GeneBank	XM_002267100.1	VvPSL1
Stem eudicotyledons				
Aquilegia coerulea	Colorado blue columbine	Phytozome	AcoGoldSmith_v1.001427m.g	AcPSL1
MONOCOTS				
Commelinids				
Brachypodium distachyon	Purple false brome	Phytozome	Bradi4g24400.1	BdPSL1
Oryza sativa	Rice	GeneBank	NM_001072397.1	OsPSL1
Setaria italica	Foxtail millet	Phytozome	SiPROV000671m.g	SiPSL1
Sorghum bicolor	Sorghum	GeneBank	XM_002450341.1	SbPSL1

ISOETOPSIDA				
Selaginella moellendorffii	Club-mosses	Phytozome	fgenesh2_pg.C_scaffold_6000273	SmPSL1
