# Supplementary figures and images for: Evolution of Parallel Spindles Like genes in plants and highlight of unique domain architecture#
Source: BMC Evol Biol. 2011 Mar 24;11:78. doi: 10.1186/1471-2148-11-78 (PMC3071787; doi:10.1186/1471-2148-11-78)

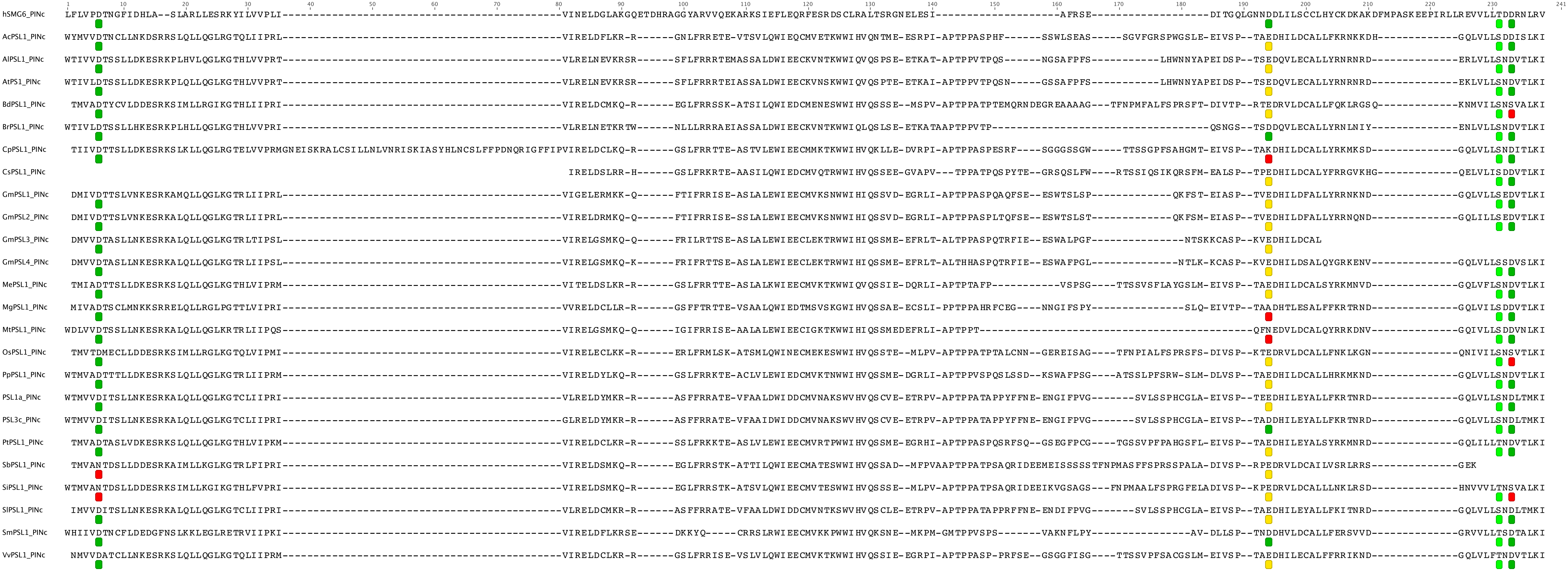

Supplement: Additional file 2 — Alignment of PSLPINc and hSMG6PINc residues. The protein domain alignment of PSLsPINc and hSMG6PINc is reported showing the conservation of catalytic residues. Dotted lines represent gaps in the alignment. Active sites are labeled with a green octagon when the residues are conserved among PSLsPINc and hSMG6PINc. A yellow or a red octagon mark an aminoacid substitution of same or different polarity, respectively. [file 1471-2148-11-78-S2.PDF]
