# Supplementary material for: Evolution of Parallel Spindles Like genes in plants and highlight of unique domain architecture#
Source: BMC Evol Biol. 2011 Mar 24;11:78. doi: 10.1186/1471-2148-11-78 (PMC3071787; doi:10.1186/1471-2148-11-78)
Supplement: Additional file 3 — Primers used in this study. Primers used for the isolation of PSL genomic clone and cDNAs [file 1471-2148-11-78-S3.PDF]

Additional Table 1 - Primers used in this study

Primer Name	Primer sequence (5'→3')	Purpose
PS_F1	ATGGCGGAAAAGCAAGAAT	
PS_R1	TTGGAAATCTTCTGCTGTCTCA	Cloning of *PSL1* genomic sequence
PS_F2	GGCTTAGCAAGTTCCACAGG	
PS_R2	TTTGACATCTTTGATAAGAAGGG	
PS_CF	CCTCTGTCTCTTTCTCTTCTCTCTCCC	Cloning of *PSL* cDNAs
PS_CR	ACCTAGCTAACTGTGCTGCCTGA	
